# The Entanglements of Affect and Participation

**DOI:** 10.3389/fpsyg.2019.02815

**Published:** 2019-12-13

**Authors:** Pirkko Raudaskoski, Charlotte Marie Bisgaard Klemmensen

**Affiliations:** Department of Communication and Psychology, Aalborg University, Aalborg, Denmark

**Keywords:** affect, emergence, disability, participation, relationality, social practice

## Abstract

The purpose of the article is to elaborate on the scholarly debate on *affect.* We consider the site of affect to be the activities of embodied, socioculturally and spatially situated participants: “Affective activity is a form of social practice” (Wetherell, 2015, p. 147). By studying affect as a social phenomenon, we treat affect as a *social ontology.* Social practices are constituted through *participation* in social interaction, which makes it possible to study affect empirically. Moreover, we suggest that to consider affect a social ontology connects affect to *agency.* We regard affect as a participants’ phenomenon where emotions and knowledge are not separated, i.e., as a *social epistemology*. To capture the complexity of affective activity, the study of situated participation requires video data. We collected data at a center for persons with acquired brain injury (ABI), which highlights research *ethics*. The International Classification of Functioning, Disability and Health (ICF) framework defines participation as involvement in life situations. ICF focuses on two broader perspectives: the body and the individual in society. We turn ICF’s abstract societal perspective on participation to meaningful local accomplishments in lived social practices. Our focus is, in line with a critical social ontology in disability studies, on *how-ability*, the communicative abilities of the residents (Hughes, 2007). To get closer to life situations as they unfold, we analyze participation in its details as embodied actions during activities in the material environment of the center. To conclude, we demonstrate a resident’s competent participation in an occupational therapy session through a fine-grained analysis of affective activity. Interaction, practices, and phenomena are complex theoretical and practical issues. In the analysis of the encounters as complex multimodal and -sensorial situations, we use an extended version of ethnomethodological conversation analysis (EMCA) that incorporates the body and material environment with the interconnectedness of interactional episodes. To do this, we enlarge the scope of analysis from the complexity of local occasions of affective activity to connections between consecutive affective entanglements. In the indicated work we draw on theoretical (*lamination*) and methodological (*nexus analysis*) suggestions in order to best pursue the sociocultural nature of situated interactions.

## Theoretical Framework

The International Classification of Functioning, Disability and Health (ICF) framework defines participation as involvement in life situations. ICF focuses on two broader perspectives: the body and the individual in society (World Health Organization [WHO], 2001, 2013). However, ICF lacks the important perspective of local social interplay of participation. The main purpose of the article is to provide an example of a method that can empirically capture the central postulate of affect as social practice: How here-and-now affective activities, including practices, places, and persons are entangled with the past. By studying affect as a social phenomenon, we treat affect as *social ontology.* Social practices are constituted through *participation* in social interaction, which makes it possible to study affect empirically. We hope to contribute to the ongoing discussion about methodology, with two main purposes. First, we aim at creating a deeper awareness of affect as social practice, that is, as local, embodied participation with intended or unintended consequences. Second, we hope to inform the methodology of affect studies. This is why we at the end of this article give an illustrative analysis of embodied participation in social encounters. The analysis can be enhanced by considering social encounters as complex, emergent multimodal and -sensorial situations that are entangled with “larger” sociocultural meaning making. Furthermore, we illustrate how language, body, and the material environment are used as resources in these entangled affective activities.

The “turn to affect” that has prevailed in the theoretical discussion across fields in humanities and social sciences, psychology included, has started a methodological debate based on the ontological nature of affect. One direction considers affect as something that is hard to detect with traditional “representational” methods (e.g., Blackman and Venn, 2010) whereas the other direction argues that affect can be regarded as an embodied undertaking by participants in ongoing social practices (Wetherell, 2015). We tackle affect as an inherent part of the activities of embodied, socioculturally and spatially situated participants: “Affective activity is a form of social practice” (Schatzki, 2001; Wetherell, 2015, p. 147). We find that this interest coincides with the paradigmatic developments, not least among feminists, that focus on relationality and materiality. For instance, Barad (2007) and Haraway (2004) wrote about the ongoing realization of the world that is entangled with a plethora of other people and entities, situations, and places, both past and future. We want to take these theoretical premises as our point of departure when we study the participation of residents with acquired brain injury (ABI) from the point of view of their competencies, that is, as *how-ability* (Raudaskoski, 2013) and *integrational proficiency* (Harris, 2009; cf. Klemmensen, 2018). We find that our take on the affect turn resonates well with critical social ontology in disability studies (Hughes, 2007). Following this, we will empirically illustrate the potential of persons with ABI through a close analysis of competent participation by a resident who challenges the ongoing reality production (cf. Potter, 1996) through affective activity. Therefore, not only do we want to exemplify a methodology for undertaking affect studies but also hope to contribute to the development of tools to investigate disability and care. Advancements in disability studies help enhance the quality of life of impaired individuals through awareness of the socio-affective consequences of social practices.

As communication scholars, our empirical research interest in situated action as emergent and entangled matches the theoretical interest of affect scholars within psychology. We put forward a possible way to analyze evolving interactions as *assemblage* and *emergence*, the central concepts of the affect turn, also in psychology. With assemblage, the nature of affect as a complex relational phenomenon is accentuated, as it includes a multitude of effects of past assemblages. With emergence, the processual aspect of the ongoing situation as an assemblage drawing on past assemblages is foregrounded (cf. Wetherell, 2015). There seems to be two different foci in understanding the connection between affect and assemblage in psychology. One approach regards the ongoing intertwining of bodies, practices, and timescales as assemblages of internal bodily sensations (Blackman and Venn, 2010), whereas the other approach considers assemblages as detectable in social practices (Wetherell, 2012). In the latter approach, participants express themselves through embodied and discursive action as other-oriented beings and interpret others holistically; they exhibit situated social epistemology.

## Affect as Social Ontology and Epistemology – Consequences for Methodology

In our view, affect as social ontology helps depict the subtle consequences of psychologically, materially, and temperospatially shared aspects of human interaction. In cultural psychology, a central interest is processuality that manifests in social practices: “Descriptions, accounts, narratives, and other kinds of discourses acquire a substantive role in the recursive constitution of diverse social practices (that is to say, in its ontology).” (Campos et al., 1999). However, we find that the focus in cultural psychology has been more on individual sayings and doings, rather than the interactional constitution of those practices.

The psychologist Lisa Blackman criticizes a discursive approach to affect and emphasizes affect as an entangled bodily phenomenon; the starting point is the internal experiencer. Both Blackman and the discourse analyst and social psychologist Margaret Wetherell regard the entanglement of emotion, and thus affect, as a habitual and shared inclination (cf. James, 1950). Notwithstanding this, recent studies emphasize the complexity of the connections between people, pasts, encounters, and materiality as emerging through participation in the situation at hand, and its unique situatedness in time and space (Wetherell, 2012; Blackman, 2013). Blackman and Venn (2010) foreground that a new ontology is on the rise, dismissing the strict division into different scientific and scholarly fields. We are in the beginning of an epistemological shift toward challenging the traditional polarities, for instance, the social and the natural, and the cognitive and the affective [cf. Barad’s (2007) ethico-onto-epistem-ology]. This paper contributes to the ongoing quest to come up with research methods that match these openings. One way to tackle this is through multimodal interaction studies of situated participation (cf. Wetherell, 2013). We do this from the perspective of “social,” as we concentrate on how to empirically capture the subtle influences of the past and present in the complexity of social practice. Analytically this means that we start with the event, rather than affect (Wetherell, 2015):

Rather than affect *per se* on a pedestal, as the topic, we can become interested in a multi-modal situated event, in a consequential set of sequences in social, cultural, and institutional life, and make connections between the emotional performances and other ordering and organizing constituents. (p. 159)

We argue that we can answer Wetherell’s call by studying affect with a methodological framework from practice and interaction studies. This allows us to investigate the complex assemblage or entanglement of emotion, materiality, and historicity in a study focusing on situated, multimodal meaning-making practices. In sum, we consider affect as social and embodied when it travels as a bodily phenomenon (Blackman, 2012, p. 15). As mentioned above, our situated doings are always an assemblage of past, present, and future. Wetherell’s idea of a practice-oriented affect focuses on the many past influences present in a situation. We combine this position with ethnomethodology’s focus on social order as a local accomplishment (Garfinkel, 1984) in which the past is present implicitly through the participants’ understanding of and acting according to the *gestalt* they figure any situation to be (see e.g., Emirbayer and Maynard, 2011). To be able to do this, the participant has to have previous experiences with similar situations. Thus, we consider affect an inherently relational phenomenon that is detectable within and between situations of social interaction (e.g., Raudaskoski, 2017b). The ongoing social practices witness the affective labor with which the participants show their (dis-)affiliations in the situation as a sense-making event. It is this social epistemology, the ongoing constitution of correct versions of the world, that we try analytically to capture in its details.

## Affect as Emergence and Assemblage – Methodological Consequences

Both Blackman and Wetherell call for an interdisciplinary approach to investigate human interaction as *practices* of meaning-making by shifting focus to a perspective which foregrounds emergent, inherently indeterminate multimodal and -sensorial action organized as emergent assemblages. What connects Blackman and Wetherell is that both refer to an understanding of affect as a complex assemblage. Wetherell affect’s assemblage is more like Latour’s use of it (e.g., “action at distance”; how materialities and practices from other times and places influence the present), while Blackman resorts to an earlier, in psychological research dismissed, version of “action at distance” that is even harder to show empirically (e.g., telepathy). Blackman (2008) resurrects Bergson and other vitalists in the relational affect studies of experience (cf. Brown and Reavey, 2015). Blackman criticizes science studies and cultural theory for treating the body as a separate neurobiological entity to be affected. This understanding of body detaches the emergent aspect of affect and campaigns fixity rather than plasticity and emergence (Blackman, 2013, pp. 196–197). Blackman (2008) demonstrates how sense-making has been traditionally conceptualized as a work of thought and talk, and, therefore, the importance of embodiment and action has been overlooked. One of the authors of the present article has pointed out earlier how this inclination toward mind–body duality has been also problematized in bordering fields (e.g., Enfield and Sidnell, 2017; Hutton, 2017). Of greater importance is the notion of integration of past–present–future: A distinct human feature, according to Harris (2008) conceptualized as an activity, and not a work of thought:

Everything we do as human beings involve the integration of the present with the past and the future: this is temporal integration. The past we can only remember and the future we can only anticipate. But unless we could relate the here-and-now to both of these, our lives would not be those of human beings (.) human beings communicate with one another not by exchanging thoughts but by integrating their many activities. (p. 111)

Even if the importance of haptic perception, actions, and the body are increasingly in focus in processual meaning-making studies, talk and sequentiality still tend to be the methodological focus (Klemmensen, 2018). Klemmensen (2018) claims that linguistic competency is the focus in most logopedic studies with impaired individuals. However, studies in affect would suggest practices, emergences, and entanglements as social ontology and preferred analytical focus. In line with the position put forward with critical social ontology in disability studies, we consider it more ethical in the study of vulnerable subjects to broaden the perspective and have embodied action more in focus.

Important for our methodological considerations, Wetherell (2015) argues that affect is occasioned (plasticity and flexibility) and it is historical, encompassing “the human work involved in being emotional and being affected, in parsing and categorizing affective states, and the exquisite, highly complex intersections between body states, methods of registering and describing these, and the context.” (p. 146). In an empirical study of affect, the assemblic and intertwined nature of emergent practices demands more careful attention than, for example, tracing the various developments and formations of activity types or their resources in longitudinal EMCA studies (Doehler et al., 2018). This is why we situate our approach to affective methodology in an interdisciplinary field informed by interaction studies and practice studies, the interest of which also lies in the manifold connectivities between practices. Since EMCA requires proof for any analytical claims from the data, affect as interpersonal emotion or narrative is mainly researched through the sequential responses to a participant’s talk and action, instead of making claims and guesses about the intention of the speaker. A fairly recent collection of papers with an EMCA approach to emotions as embodied actions can be found, for example, in Peräkylä and Sorjonen’s (2012) edited collection. Affective activity includes clear emotional displays, but the social practices that can be considered and analyzed as affective experiences. These can be of more varied types such as diversely expressed intensities in interaction.

To study affect as the subtle influences of past and present in the complexity of social practice requires a further development of methodologies that appreciate the situated accomplishment of action as an assemblic undertaking. Affect is not an ephemeral entity, but constantly configures and reconfigures actions as they unfold. In other words, affect is entangled with participation, which “refers to actions demonstrating forms of involvement performed by parties within evolving structures of talk.” (Goodwin and Goodwin, 2004, p. 222). Wetherell (2013) takes Goodwin’s (2006) work as a prime example of how to trace affect in social interaction. Marjorie H. and Charles Goodwin were among the first in the EMCA community that understood the significance of not just sequential analysis, but the analysis of the participants’ actions as embodied, often simultaneous undertakings with talk, in material environments. For instance, Charles Goodwin demonstrated already long time ago the importance of analyzing contributions to interaction as relational, as being shaped by the other participants, also during a participant’s contribution (Goodwin, 1979). We apply their extended version of EMCA that incorporates the body and material environment with the sociohistorical nature of interactions in order to approach encounters as complex multimodal and -sensorial situations. To offer a methodology that serves affective activity as both emergent and assemblic, we find it necessary to enlarge the scope of analysis from the complexity of local occasions of affective activity to connections between consecutive affective entanglements. EMCA studies rarely pursue an analysis of the sociohistorical nature of situated interactions. Since we explore an interdisciplinary field, we study not only close, multimodal, and nuanced analysis of affective activities (cf. Goodwin et al., 2012; Wetherell, 2015; Goodwin and Cekaite, 2018) but we also trace their mutual connections over time. For the present paper, this enables us to shed light upon how-ability (Raudaskoski, 2013) of the participation by impaired individuals as competent laminating to the ongoing overall activity, which means that we also analyze their observable integrational proficiency (Klemmensen, 2018).

## Methodological Entanglements

We want to trace affect from local complex entanglements in the evolving interactions. However, instead of only closely examining various episodes of talk and action as evidence of affect, we want to see how various episodes connect in order to open up the theoretical entanglements for our analytical gaze. As indicated above, we prefer Wetherell’s methodological approach to affect as it presupposes empirically observable social practices and encourages to follow affective activities as they are formed through bodies in social interaction with each other and the material environment. Goodwin (2013, 2018) work captures the processuality of the material–semiotic environment. Its description of the emergent entanglement (lamination) of “materials” in interaction has theoretical connections to the notion of ontology in practice theory (Schatzki et al., 2001). Goodwin defines “materials” with a sociocultural understanding as entities from the past, whether the immediately preceding one (e.g., turn-at-talk) or (tools) from other time-spaces (see the next section for a more thorough introduction to the concept of lamination). The notion of *contextual configuration* (Goodwin, 2000) helps analyze the moment-for-moment composing of these materials in practical action. As discussed by Klemmensen (2018), practice theorist Schatzki’s inclination toward Heidegger and Wittgenstein’s ideas of emergence allows a close description of multiple timescales formulated as “indeterminacy” in social events (Schatzki, 2013). This view of the social event as an endless multiparty concerted semiosis of social practices is in accordance with both Goodwin’s and Schatzki’s view of social events as situations emerging from certain pasts and being under construction, in other words, indeterminate social actions. According to ethnomethodology, indeterminacies get temporarily fixed in the unfolding action for the participants to be able to do things in practice.

Schatzki (1997) advocates a practice agenda in social ontology, which fits well with our framing of affect as social practice and, therefore, participation. Schatzki’s (1997) concept of *teleoaffectivity* accentuates the ongoing relevance of any practice:

By teleoaffectivity, I mean orientations toward ends and how things matter. What a person does is largely dependent on the things for the sake of which she is prepared to act, how she is oriented toward proceeding for them, and how things matter to her. (p. 302)

Schatzki (2001) discusses social practices from the perspective of teleoaffectivity, as always being evaluable in relation to their acceptability or correctness. Furthermore, the ongoing evaluation of concrete action as acceptable or not comes close to an ethnomethodological understanding of morality and norms as ongoing accomplishments that can, therefore, be regarded as affective activity.

Wetherell’s (2013) affect stance also draws from practice theory’s processual focus. Our methodological choice comes very close to that of Wetherell’s yet takes it further by concentrating more on the intricacies of analysis. Especially, the details of the rhizomatic nature of the entanglements draw our methodological attention. Affect becomes observable in people’s participation, in their interactive work. In the present data we concentrate on special cases of teleoaffectivity – how *counterclaims* are managed as participation concerning mattering and acceptability. Wetherell’s position makes it possible for the present authors to approach affect from their two slightly differing foci on interaction and meaning making in general: (1) trying to understand an individual’s experiences (Nielsen, 2015; Klemmensen, 2018) and (2) trying to understand affect as an embodied, place-based, nuanced practice (Raudaskoski, 2010, 2016, 2017a, 2018). Klemmensen (2018) has introduced an interdisciplinary perspective to the tracing of practices over time in an analysis that outlines a person-centered approach to interaction with aphasia and ABI. Raudaskoski (2010) has analyzed affect as social practice from a telephone call about a child-in-referral to adoptive parents. Her papers from 2016 and 2018 extend the discussion on affect with a special reference to Blackman and Wetherell in relation to sociomateriality (also developed in 2017b; video data from a nature hike), and in relation to imagination, morality, and norms (video data from two TV interviews).

In sum, our methodology incorporates Wethrell’s approach to affective activity as social practice with Schatzki’s (2002) practice theoretical definition of teleoaffectivity and Goodwin’s co-operative action. Schatzki’s approach is helpful in grasping how affect is entangled with past actions and the assemblic present. However, it does not provide an interaction-based methodological framework to analyze teleoaffectivity as a phenomenon of situated “site of the social” (2002), nor does Schatzki indicate how to follow connections. We, therefore, find it useful to combine Goodwin’s and Scahtzki’s approaches with Scollon and Scollon’s (2004) nexus analysis (NA), an empirical framework for sociocultural analysis that provides a methodology for tracing social practices.

## Laminated Action

In order to grasp better the sociocultural traceability or historicity of emergences in empirical data, we first turn to Goodwin’s concept of *laminated action* (2013):

Individual actions are constructed by assembling diverse materials, including language structure, prosody, and visible embodied displays. Semiotically charged objects, such as maps, when included within local action, incorporate ways of knowing and acting upon the world that have been inherited from predecessors. New action is built by performing systematic, selective operations on these public configurations of resources. (p. 8)

Lamination covers the here-and-now, and the moment-for-moment-building of other-oriented action, but also the pasts that are present in situated action, semiotically and materially as the substrate to which the present action contributes. To study lamination, we explore how various types of doings and sayings in material settings constitute contextual configurations through various constellations of “semiotic fields” (e.g., language, body, and artifacts). By decomposition and reuse of material from previous turns, experiences, and expectations accumulate and constitute knowledge as the product of humans co-operating (Goodwin, 2018). Yet, Goodwin’s lamination functions at two, fairly separate levels: (1) the local, turn by turn building of interaction in which the previous turn can work as “substrate.” In this local co-operative building of meaning, (2) materials from predecessors point at longer timescales and practices. However, we want to inspect lamination as a phenomenon in between these two timescales as a process. We want to detect and follow the development of issues that matter as embodied undertakings. In our case an important trace is the embodied, situated (as activity and material setting) production of counterclaims. We are interested in a methodology that can follow the episodes of interactions in order to describe how they connect to each other. Thus, methodologically, we apply relationality by developing a data-driven method of tracing affective entanglements from longer stretches of interaction. This is where we turn to NA.

## Analytical Framework

In order to approach the relationality of affective assemblages, NA (Scollon and Scollon, 2004) serves as our general framework. NA shares the same theoretical and methodological interests as Goodwin’s lamination. It is an ethnographic approach that provides a possibility to combine close interaction studies with an understanding of the historicity of the ongoing action (Scollon and Scollon, 2004, 2007). NA is a framework for doing mediated discourse analysis (MDA) (Scollon, 2001), which, with its focus on embodiment and materiality has resemblances to Goodwin’s (2000) contextual configuration (cf. Raudaskoski, 2010). NA regards social actions in a situated activity (*nexus of practice*) as a most important focus. The analysis starts with nexus of practice, which often is a habitual and recognizable activity (*interaction order*) and always an intersection of place-bound (cp. Casey, 1987) discourses (*discourses in place*) and participants (*historical bodies*) – all with past histories. How far in the sociocultural past the researcher goes with data analysis (circumferencing) depends on what is being investigated. Therefore, NA – as also discussed by the practice studies researcher Nicolini (2012) – provides a *practice-based* framework for analyzing entanglements or assemblages, also affect and agency, as rhizomatic. This type of study goes beyond discursive discrepancies or interactional dissonance as strictly local occasions, and focuses on relationality and participation as consequential (Larsen and Raudaskoski, 2016; Klemmensen, 2018).

Nexus analysis, combined with contextual configuration, provides a framework that makes possible a close analysis of ongoing action with connections to other times. In sum, the enrichment is that it affords traceability by *following* the actions, not just stating the connections between them and other times. NA is, therefore, to be considered the methodological answer to Wetherell’s description of affective practice: “An affective practice like a dancing plague recruits material objects, institutions, pasts and anticipated futures. But the main things that an affective practice folds or composes together are bodies and meaning-making.” (Wetherell, 2012, p. 20).

## Empirical Data

In order to show how we have operationalized the above methodological constructs, we now turn to our illustrative empirical analysis. During 2012–2013 we carried out a pilot project about inclusion and exclusion in an ABI institution/home setting. In order to observe (and participate in) the everyday life of people with ABI, we paid a series of fieldwork visits to a care home facility in Northern Denmark. Five visits over 3 months formed the core pilot project. The pedagogical principle of the center is social inclusion (cf. ICF framework) that is conceived as the enhancement of the residents’ possibilities to be part of social situations. We wanted to research how inclusion as a popular concept in care was practiced in the center, and what kind of exclusions could be detected. In the same way as words can be ethnomethodologically seen as approximations the meaning of which is fixed *in situ*, we considered whether a social practice includes or excludes depends on its local accomplishment, and not on its intended effect. This is why the overall approach was to follow lived practices as complex accomplishments of embodied participation in material settings. Data were collected through participatory fieldwork by the researchers who, while participating in the everyday activities in the center, took notes, conducted interviews, and made video recordings (cf. Jordan and Henderson, 1995; ten Have, 2004; Raudaskoski, 2015; Demuth, 2018). Combined, the data form the “core data” and supportive evidence (ten Have, 2004). For the present article we concentrate solely on the video data, as our aim is to show how multimodal video analysis of longer stretches of interaction (Goodwin, 2013, 2018) can add to the situated analysis of affective activity (Wetherell, 2015).

Our empirical video data for the present article come from a bi-weekly occupational therapeutic meeting that took place in the Competence and Culture Center (a meeting room). In addition to one stable camera (Panasonic) in the corner, two GoPros were used, one sitting on an elevated table at the front and the other one being attached on the forehead of the cameraperson. By using three video cameras we wanted to cover as much as possible of the various participants’ communicative resources (cf. Raudaskoski, 2003). In our analysis, we follow how one resident skilfully fits his critical participation in the ongoing interaction and how he builds that affective engagement on his previous embodied or verbal contributions during the meeting. We follow him over the course of three exemplary excerpts that illustrate his habitual *modus operandi* or social behavior with the care personal and social encounters.

In line with Hughes (2007), the focus is on the residents’ social abilities, rather than on their physical or cognitive disabilities (cf. Raudaskoski, 2013). At the time of recording (2012), almost exclusively all theories and research on brain injury focused on psychological and neurological issues of the brain itself. There was very little research-based understanding of the social/communicative/interactional consequences of brain injury for everyday life, even if there was some research into the possibilities of self-presentation (e.g., Hydén and Antelius, 2011). We chose to follow what went on at an everyday level of lived practice to search for indicators of which practices were inclusive and which practices led exclusion from participation. This is why in our study the residents were followed in their everyday (institutional) environment. We had open-ended access to define our research through an institutional collaboration and were not commissioned by the board of the care center. However, we discussed our initial ideas with the pedagogical leader and his manager and held a workshop at the center to share our ideas and observations during the pilot phase where staff, residents, and administration were invited and a number of researchers partook (cf. Nielsen, 2015). We also reviewed parts of our material with the occupational therapists (OTs) and the participant residents during the pilot. Both of the authors of the present article were involved in the fieldwork. We followed the general research ethical protocols from EMCA, acquiring undersigned consent forms from all the participants or their carers (in case of severe brain injury), and the participants were informed that they can at any stage revoke their permission to use the data. The form made it possible to give a detailed permission to use the anonymized data in research and teaching with reference to the initial project. As the researchers were participating in the occupational therapy situations as interested parties, instead of trying to be undisturbing observants, they were moving about freely in the same way as the other participants were. Nothing was done to hide that research took place. In other words, objectivity was regarded as closeness, not as distance (Clarke, 2005). This is why the researchers always are participants in the situations analyzed below.

Since we consider affect as social practice in which various assemblages are present, we analyze it through emergent participation. In the following, we undertake a fine-tuned analysis of participation as embodied social practices while they unfold in their material setting (Scollon and Scollon, 2004) and also how the previous occasions are present rhizomatically, popping up from “substrates” (Goodwin, 2013). We follow the EMCA principle of *unmotivated looking* in our striving to document how exactly the participants oriented to each other and the material surroundings; how exactly did they use language, gaze, and the body, how were the ongoing contextual configurations built to show where their attention was. This we did through including longer stretches of data and investigating relationality within and between parts of these from the embodied participation (cp. Raudaskoski, 2003). So, we aim at combining the strengths of several existing approaches to action: the Goodwinian type of close EMCA analysis (Goodwin, 2003, 2013, 2018), the practice theoretical understanding of teleoaffectivity (Schatzki, 1997), and the experiential approach as historical and layered (Scollon and Scollon, 2004; Goodwin, 2013). We find in this interdisciplinary conjunction of lamination and NA a possible methodology to analyze our data as “‘composing’, ‘figuring’, ‘entangling’, ‘mobilising’ and ‘recruiting’. Something, in other words, that comes into shape and continues to change and refigure as it flows on.” (Wetherell, 2012, p. 15). The inclusion of teleoaffecivity is used as a concept that helps give an explanation from the point of view of affect as disalignments and disputes which recurrently emerge.

With the present implementation of methodology the consequences of ABI to the body and its functions are investigated in an analysis of lived practices in an institutional setting.

## Analysis of Affective Activity as Embodied Participation

We focus on one of the biweekly sessions where an OT and pedagogical staff members are always present, with a varying number of residents. The occupational therapy sessions are fairly informal gatherings without a strict procedure. The session in question took place in the Competence and Culture Center room where the residents can engage in, for instance, discussing newspaper and magazine articles or plan future activities such as shopping and local competitions. The atmosphere in the meetings we followed were generally upbeat – there was a lot of laughter and teasing. The session in focus lasted for 2.5 h. We follow a resident when he (1) volunteers to make tea in a kitchen area before the session and again (2) back in the meeting room during the introduction to our research project and, further, (3) in a discussion about the variety of ABI as a practical problem. In these episodes (a) an OT highlights the difficulty for disabled (ABI) residents to use the building’s interior design, (b) a researcher claims that people with ABI get easily tired, and (c) a discussion takes place about the center’s understanding of the various types of problems people with ABI have. In the three occasions of participation we analyze how R problematizes these claims through (a′) orienting to the skilful use of the kitchen and doing that with interactional finesse, (b′) skilfully “teaching” the researcher about his body (spastic right arm) causing insomnia, and (c′) highlighting the ignorance of the staff vis-à-vis his experiences in the place of care. The three foci emerged from unmotivated looking as an analytic strategy: We noticed that issues from the kitchen were taken up in different ways in the consequent meeting (in the second: ABI as disability; in the third: the concrete setting and care).

**FIGURE 1:**
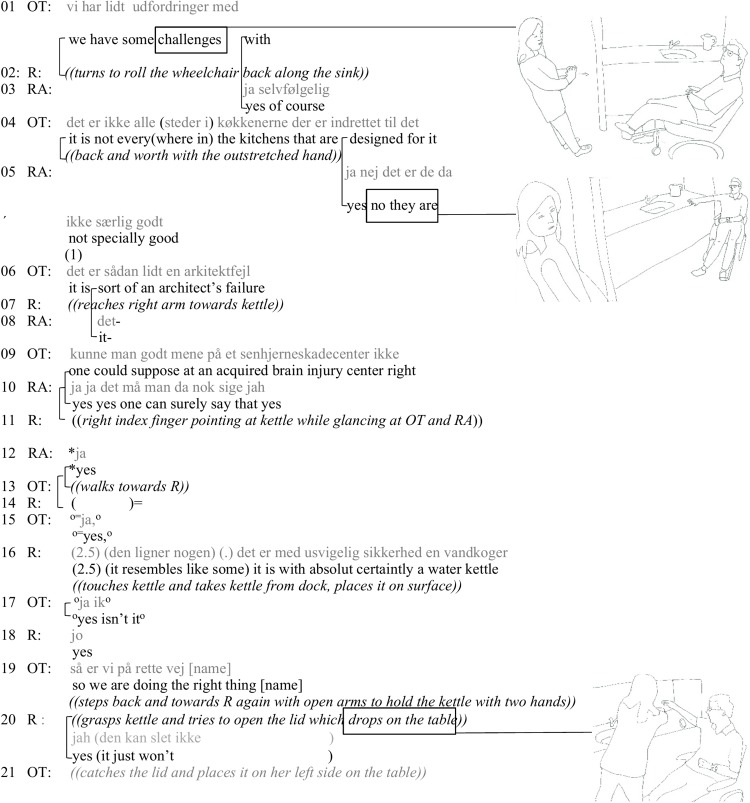
The kitchen as a difficult material setting for people with ABI.

In the following, we explain briefly what has happened before each extract. Before the first extract, the researchers have been introducing the research project in the occupational therapy room. There is coffee on the table, but tea is missing, so a resident (R), an OT, and a research assistant (RA) have moved from the meeting room to the adjacent kitchen in the common area where OT and RA have agreed to make tea with R. There is humorous talk about the RA’s headband with GoPro [cf. Murakami (2003) on the joint attention to a technological device in a data gathering session]. The transition to tea making takes place when OT places herself at one end of the kitchen sink while asking, through a hand gesture and subdued talk, R to go ahead. R starts moving to the sink in his wheelchair in a direct angle to the sink. The angle is such that he would not be able to reach the objects on the sink. Seeing this seems to occasion OT’s critique of the interior design that she addresses to RA. This is where the extract starts (Figure 1).

Through turns 1–9, the OT is verbally and through gesturing highlighting the building’s bad interior design, in relation to disabled individuals’ participation possibilities. By doing this, the OT steps out of her role of a co-participant in tea making, as she turns to the RA and “lectures” about the bad design for wheelchair users. This way she constitutes R as a member of a general collection (a person with bodily impairment) that she is talking about, and doing that in front of R. OT verbally initiates the critique in turn 1. R rolls back in his wheelchair toward the sink to initiate the activity of making tea. R’s adjusting his wheelchair to go alongside the kitchen sink is a necessarily maneuvre to reach the kettle, but as it is done in overlap with OT’s talk, it could also be seen as an embodiment of her critique (cf. the analysis in Klemmensen, 2018, pp. 121–122).

In turn 11, R reaches out for the water kettle and changes the hand shape from grasp to pointing while glancing both at OT and RA. OT, who has two participation frameworks, that of speaking with RA while the trunk oriented to R, interprets R’s pointing and glances as an attention seeking activity. She walks to R who talks to her with subdued voice (turn 14). While grasping the kettle from its base, and while glancing at the approaching RA, R discursively (turn 16) approaches the water kettle in a jokey fashion as the absolutely correct object. This way he laminates to the topic of the bad kitchen sink design for disabled people by transforming the topic “disability” (and kitchen sinks) to a humorous way of starting the actual tea making [cf. e.g., Mulkay (1988) on humor as problem hedging and Argaman (2015) on humor and disagreement]. We find this change of topic his way of getting the focus on him as skilful in not just how to use the (objects on the) kitchen sink, but also in his linguistic and interpersonal abilities, shown in a complex way of expressing a humorous stance. OT joins the humorous line by mock treating R’s turn as information to acknowledge, telling R that his trajectory of action is correct with the kettle. While saying this, OT shows her professional orientation to the situation as practizing everyday life skills, and steps back. R has difficulties getting the lid off the kettle, and OT steps forward toward him once again (turn 19). While she is holding the kettle, R attempts to get the lid out and comments on how it will not release (20). On the cooperative use of material objects, see Raudaskoski (1997, 2000, 2003, 2006) and also the recent interest in EMCA on the topic (Nevile et al., 2014). After that, the lid gives in and drops on the surface of the sink. In duo, R and OT handle the kettle co-operatively in a co-choreographed fashion that allows R to participate in the tea preparation gradually, laminating each other’s actions over turns 11–21. So the difficulty for R in the situation turns out to be – due to his spastic left arm – his inability to use both hands to get the lid off, rather than him being in the wheelchair.

Affective activity as social practice in this extract is subtle: the resident challenges the categorization made by the occupational therapy. He does that through bodily action (changing the angle of the wheelchair to the kitchen sink) and by participating in the situation in a humorous fashion. His agency is only limited by the spastic left arm.

**FIGURE 2:**
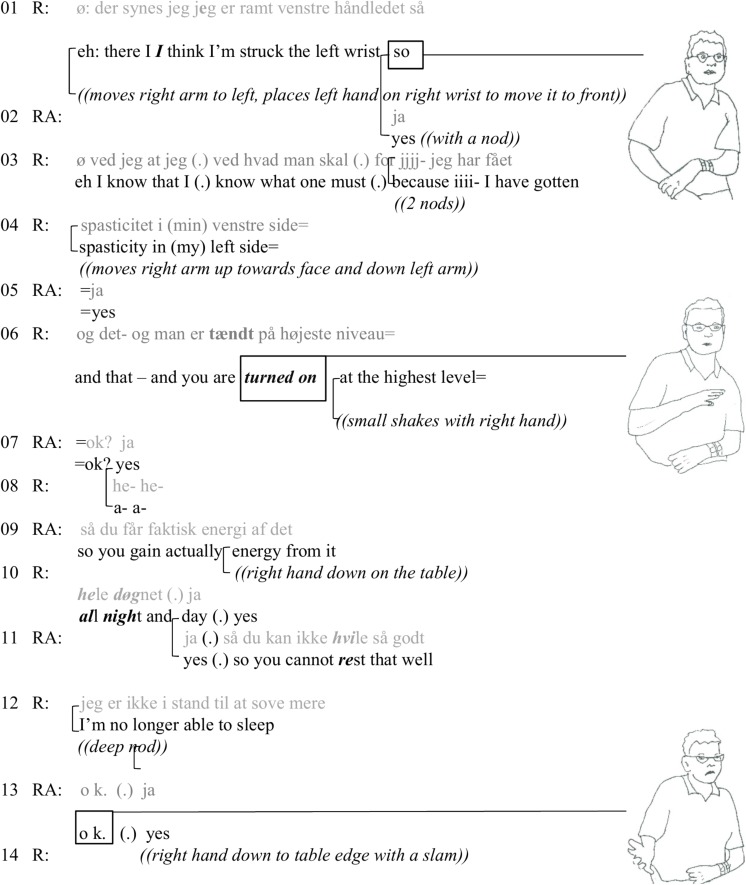
The resident’s body (spastic right arm) causing insomnia.

The next extract comes from the introduction to the research project in the meeting room. RA has given each participant a sheet of paper, which explains the project and its purpose. She is standing up and reading the letter through, explaining some of the sentences with her own words. Just before the second extract (Figure 2) she explains – through her own experience with a family member that has had strokes – how very tired a person with ABI easily gets. R’s participation is a reaction to that.

In this excerpt the resident (R) makes a counterclaim to RA’s generalization about ABI as always causing tiredness. R starts his respond by locating his bodily problem first to the left wrist and then expanding it to spasticity in the whole left side (turns 1–4). He moves the left lower arm to the front of his body by grasping the right arm with it and moving the right arm to his side. He then releases the right arm to move it down the left arm when he talks about the left side (see picture in turn 1). This bodily problem is then turned into a description of his bodily state (“turned on”) in turn 6. RA is showing her understanding of the description as “getting energy” of the bodily impairment by her change of state token and affirmative feedback (turn 7). She then gives a formulation (“so you gain actually energy from it”) of how she understands R’s contribution. R’s next turn (“all day and all night”) is at the same time a continuation of his first turn and an acknowledgment of RA’s formulation. RA now formulates the gist of R’s further explanation (turn 10) with “so you cannot rest very well,” to which R agrees with a more extreme case formulation of no longer being able to sleep.

Resident builds his counterclaim carefully. Instead of telling RA that her generalization is wrong, he builds his case about his body with his body; he laminates the talk about the problematic part of it with a demonstration or visualization. The problem with the left arm already had become noticeable with his difficulty to move the water kettle lift by right hand only. His “diagnostic work” (cf. Büscher et al., 2010) could be seen to laminate to that occasion, too, and not just as a preparation to adjusting the claim that RA had made. By introducing the problem with the left arm and by letting RA formulate the contradictory point of view (“you cannot rest”) to her previous announcement of being extremely tired, R is being highly pedagogical and, therefore, a skilful “informant.” There are small acknowledging voices in addition to RA’s empathetic agreement. The mood is sober.

In this extract, the affective activity is more in line with the traditional focus on private feelings as shareable emotions. The resident incrementally corrects the RA’s category-bound generalization of ABI always meaning tiredness (teleoaffectivity) to him not being able to sleep because of the spastic arm.

**FIGURE 3:**
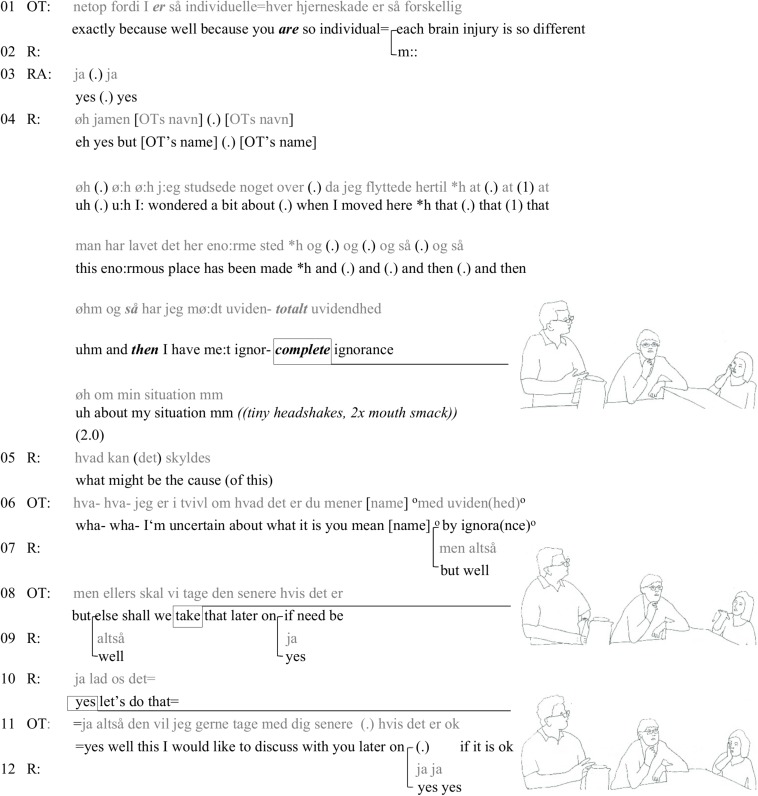
The problematic professional discourse of the site.

After this, the talk goes to discussing how each and every person with ABI is a specific case. There is a long episode of talk by the researchers and staff members about each case being different, how there is no one type that people can be categorized into. After the general agreement about each individual case being different OT relates it back to “this place here” (“this is why we define this place as a specialized residence”). The first turn in the following excerpt (Figure 3) continues from this statement, giving her reason for it.

In turn 1, OT connects the general discussion about each brain injury being different to the residency they live and work in. R turns to OT, calling her by her name, and starts a counterclaim in the form of a complaint (cp. Klemmensen, 2018, p. 123). R builds his complaint by giving an account of his first thoughts when he arrived to the “enormous place that has been built” in his extensive turn (turn 4), laminating his turn-at-talk with that of OT’s mention of the place. He then contrasts the sophistication of the building with a lack of medical understanding of his condition. He can be seen to laminate to the situation in the kitchen (Figure 1) where OT criticized the interior design of the building: It is not just the building but the care given in it that is under criticism. We can detect the nervousness (intensity) of his participation in his small headshakes and the smacks that are hearably produced in a dry mouth (turn 4).

Resident is using the highly charged word “ignorance” to describe the institutional knowledge about his situation. R’s contribution also laminates to the refuting of RA’s claim in the previous discussion (Figure 2). The general formulation of “my situation,” together with the extreme case formulation “complete ignorance” seems to throw OT off guard: “wha- wha- I’m uncertain what it is you mean by ignorance [name]” (turn 6). Right after OT addresses R, R starts answering the question (turn 7), which he repeats (turn 9) after OT’s subdued finishing of the sentence “by ignorance” and starting a new sentence (turn 8). In turn 8, OT suggests a postponing of the discussion, accompanied by a rapid movement of hand downward (turn 8, picture). The discarding of R’s intense contribution gets a minimal response from him (“yes” in turn 9). After OT’s additional “if need be,” R gives a wordier confirmation, with a pointing hand (turn 10, picture), implying his eagerness to continue with the topic. OT’s last turn (11) works both as a promise to not to drop the topic and not to do it in the present situation, categorizing it as not suitable or relevant for the situation at hand. After this episode, OT turns her gaze away from R. There is a fairly long silence (7 s) in relation to the pace of the interaction so far. The long silence not only ended the topic of the discussion in this continuously sustained talk (Schegloff, 2007), but the length of it confirms the exchange as a disruption to the ongoing topic. The silence is then broken by another staff member who changes the trajectory and starts talking about the practicalities of the research project.

In this extract, we have a counterclaim the production of which is a teleoaffective activity that is accompanied by the kind of affective activity that Blackman and Venn write about: The intensity of the resident’s feeling is not just expressed through words, but through gestures, head shakes, and the hearably dry mouth.

We have now followed three occasions where the resident orients to (a′) his ability to use the kitchen as a material and social space, (b′) his body (spastic right arm) causing insomnia, and (c′) the ignorance of the staff vis-à-vis his situation. All these embodied or verbal statements occur as next turns to (a) an OT’s highlighting of the building’s bad interior design, occasioned itself by the resident being in the kitchen, (b) a researcher’s claim of people with ABI getting easily tired, and (c) a discussion about the organizational understanding of the various types of problems people with ABI have. The three extracts give examples of teleoaffectivity: the acceptability or correctness of the claims are challenged. In the first extract (Figure 1), the problem of the kitchen as a concrete space for ABI sufferers is challenged by the resident’s humorous response, showing his social capacities, while he is parking his wheelchair to start making tea. However, his participation becomes cumbersome due to a spastic arm. In the second extract (Figure 2), the resident recruits his spastic arm as a concretization of the counterclaim to RA’s claim about tiredness. In the third extract (Figure 3) the ignorance (cf. Figure 2) of the carers is laminated to the “fine” building (cf. Figure 1).

In doing this analysis, we have shown an example of how a nexus analytical framing – benefits from an interdisciplinary methodology to study affect as social practice. NA can show at the emergent social practice level how the conduct of individuals and collectivities emerges in an entangled fashion. This is possible because NA moves the analysis across time and space in both a forward and backward perspective, instead of being sequentially restricted as are traditional EMCA analyses.

## Discussion

Affective activity is theorized as an emergent, entangled activity where the body, the material setting, the activity, and the sociocultural pasts of those intersect in emergent interaction (cf. Raudaskoski, 2003; Krummheuer, 2015; Klemmensen, 2018). The data material shows how relational affective activity develops through a series of engagements between participants. The analysis illustrates how lamination and teleoaffectivity can be used to analyze participants’ participation and initiatives in interaction. We show how affect as social practice unfolds as an assemblage or entanglement of not only the ongoing talk and action with the complexity of the embodied, material, and verbal situation, but also past occasions of participation. Therefore, the study examines affect as practice over time, demonstrating its rhizomatic connections between three counterclaims. The extracts show how an “emotional blister” (Wetherell, 2012, p. 70) grows throughout well-meaning institutional interaction when the resident (R) produces three separate occasions of counterclaims.

Our paper takes its point of departure in Wetherell’s (2015) recent acknowledgment of EMCA-based analysis of social practices as a way to do affect analysis. With this turn, Wetherell, who started discursive psychology with Potter as a critical discursive approach with interpretative repertoire as a main analytical tool, now comes closer to the mainstream interests of discursive psychology (e.g., Wiggins and Potter, 2007). However, while mainstream discursive psychology has kept close to the linguistic conversation analysis in its focus on the verbal production of talk as social action, Wetherell opens to more nuanced tools (Goodwins’ work). Furthermore, she appreciates the theoretical considerations of affect that Blackman and Venn exemplify, but is hesitant about how the theoretical focus on intense bodily experiences can be turned into an empirical analysis. For instance, Wetherell (2012) highlights that the Deleuzian concepts of affective experiences such as force and intensity are analyzed in unaccountable ways. Notwithstanding this, our analysis shows how intensity can be analyzed as part of social practice. In the last example (Figure 3) the nervousness of the resident is detectable in his embodied participation: the small headshakes and dry mouth, giving a practical example of how affective bodily reactions not always are invisible, but are occasioned and detectable in embodied interaction. Also, even if the body is important for understanding affect, Wetherell (2012) finds the focus on the body as a non-conscious immediate entity excessive (p. 35). However, in our case the body does “come first,” but not as an internal experiencer. Instead, R’s spastic left arm becomes a topic, an issue from not being able to do a practical task (open the lid of a water kettle) to challenging outsider’s generalization of ABI and tiredness (cannot sleep) to making a complaint about the institutional care.

To sum up, the scope of the present paper has been twofold. First, it is an example of affective activity as embodied participation and, second, it is a response to the call of empirical investigations on affect and does that from a multimodal and ethnomethodological perspective. It demonstrates the omnipresence and various (subtle) forms of affect in social practice. In this way, real life communication situations are shown to be essential data to study not just affective activities, but also how bodily impairment demonstrates in institutional settings. The analysis showed not just how the resident tied to the topics of the ongoing interaction, but also laminated to the previous ones, and, finally, to a memory of his first encounter with the place. The place as a material entity, both as a topic and as a setting of the interaction, was thus laminated to his managing a change of direction in the discussion. It demonstrated the complex relationality of interaction practices and especially counterclaims as teleoaffective activity. In other words, with a nexus analytical framing: The occurrence of cascading responses distributed over several situations shows how a resident (R) uses his experiences (cp. historical body) throughout sessions involving the researcher (RA) and OT (cp. interaction order), connecting the topic about the interior design of the institution (Figure 1) to a complaint about his treatment there (cp. discourses in place) (Figure 3).

## Conclusion

Our objective has been to contribute to the methodological toolkit for empirical studies of affective activity, with data from a disability context. The theorization of affect as entanglement and emergence inspired us to investigate affective activity as lived practice. We started with a discussion about two diverging approaches to affect within psychology. The first considers affect a non-cognitive bodily phenomenon (e.g., Blackman and Venn, 2010), while the second conceptualizes affect as emerging social practice (e.g., Wetherell, 2013). We chose the side of “social” in the debate between their positions.

By treating affective activity as a form of social practice in situated human interaction, we considered affect as a social ontology A social ontology also included investigating the social event as an assemblic movement across sequences. In that work we resorted to a combination of methodological tools from ethnomethodology and practice studies guided by a nexus analytical framing, which uncovered the experiential as situated place-based, material, sociocultural participation: we used contextual configuration, lamination, and NA. With these methods, the subtlety of affect could be demonstrated in an illustrative empirical analysis as a social, bodily phenomenon which is entangled with practices over time and space.

In sum, the complex theme of affect as emergent social practice requires a methodology with which the converging theoretical interests of different traditions can be served and the entanglements of affect and participation can be empirically researched. We find it important that we undertake research that can help not just understand affect and participation as theoretical or empirical questions, but can contribute to things that matter. Our empirical analysis was an attempt at that: We showed the skilful or proficient use of initiative and memory by a resident in a care center for ABI where many are diagnosed as having problems with exactly those. A single-case design is idiographic in content but with the fine-grained analysis of one participant, we show not just this particular resident’s skills but how the situations studied are entangled with various pasts, present, and future anticipatory participation.

We have shown with an analysis of a resident’s affective activity as social practice how inclusion and exclusion are not either or phenomena, but always recurring and accomplished through occasioned participation in the ongoing flow of institutional practices. Thus, in addition to exemplifying a methodology for doing affect studies, we aim to contribute in the development of tools to investigate disability and care and hereby enhance the quality of life of impaired individuals through awareness of the socio-affective consequences of social practices. We hope that increased attention toward interactional accomplishments will help develop our understanding of disability and its many social aspects (Raudaskoski, 2013; Krummheuer, 2015; Klemmensen, 2018). This is in accordance with the ICF-model from the WHO that promotes a disability conceptualization that focuses on participation, and, finally, it invokes a societal understanding of disability beyond a bio-based deficiency perspective (World Health Organization [WHO], 2001, 2013; Klemmensen, 2018, p. 32).

## Data Availability Statement

The datasets for this manuscript are not publicly available because to protect the anonymity of the participants. Requests to access the datasets should be directed to the Aalborg University Research Integrity Office.

## Ethics Statement

At the time of the study, an approval from an ethics committee was not a requirement. The study was conducted according to the Aalborg University research ethics guidelines and the Danish  data protection regulations. Written informed consent was given from all the participant subjects in accordance with the Declaration of Helsinki. Further details of the ethical procedure are explained in the article. Furthermore, legal bystanders validated consent given by those individuals that were legally deauthorized.

## Author Contributions

The authors contributed in collaboration to the writing of this article on the basis of their earlier research on the topic.

## Conflict of Interest

The authors contributed in collaboration to the writing of this article on the basis of their earlier research on the topic.
